# Genetic Diversity, Biofilm Formation, and Antibiotic Resistance in *Listeria monocytogenes* Isolated from Meat-Processing Plants

**DOI:** 10.3390/foods14091580

**Published:** 2025-04-30

**Authors:** Miguel Romeo, Amaia Lasagabaster, María Lavilla, Félix Amárita

**Affiliations:** AZTI-BRTA, Food Research, Parque Tecnológico de Bizkaia, Astondo Bidea 609, 48160 Derio, Spain; mromeo@azti.es (M.R.); alasa@azti.es (A.L.); famarita@azti.es (F.A.)

**Keywords:** *Listeria* spp., *Listeria monocytogenes*, biofilm, serogroups, antibiotic multi-resistance

## Abstract

*Listeria* species are ubiquitous microorganisms that can be present all over the food chain. They can survive under adverse conditions and are frequently found in food-processing plants. In this study, 19 *Listeria innocua* and 19 *Listeria welshimeri* strains were isolated from meat product manufacturing companies in Spain, and biofilm formation capabilities were analyzed. In addition, 37 *Listeria monocytogenes* strains were also isolated, and their genetic diversity, biofilm formation capabilities, and antibiotic resistance were analyzed too. The species distribution was similar between two food-processing plants in the Basque Country, while it demonstrated significant variation when compared to three other plants from the Valencian Community, Catalonia, and Andalusia. Biofilm formation was significant at both 25 °C and 37 °C, with *L. monocytogenes* showing strong biofilm formation capabilities. Biofilms enhance the ability of bacteria to persist on surfaces and equipment. *Listeria monocytogenes* serogroup analysis indicated significant differences between Basque Country strains and those from the other regions, with most strains belonging to serogroups commonly associated with human listeriosis cases. Antibiotic multi-resistance was a common feature among *L. monocytogenes* strains. The presence of different antibiotic multi-resistance profiles and strong biofilm-forming capabilities highlights the importance of stringent hygiene and monitoring practices to prevent the spread of *L. monocytogenes* in the food chain and avoid food-safety threats and public-health issues.

## 1. Introduction

*Listeria monocytogenes* is a Gram-positive bacterium widely distributed in the environment. It is not difficult to isolate this microorganism from soil, vegetation, or water. Given its ubiquity, its presence on farms, livestock, and, therefore, in the food chain is not unusual. It is a significant and concerning foodborne pathogen due to its ability to survive and grow under adverse conditions, such as low temperatures, acid-to-alkaline environments, and high saline concentrations [1]. The metabolic capabilities of *L. monocytogenes* allow it to prevail in restraint conditions for the growth of other microorganisms. Ready-to-eat foods are commonly associated with listeriosis outbreaks, and there is also high susceptibility in dairy products and meat derivatives [2,3].

Regarding its pathogenicity, *L. monocytogenes* is known for its ability to cause listeriosis. The infection usually manifests one of the following two forms of expression: non-invasive gastrointestinal listeriosis and invasive listeriosis. The non-invasive form typically results in mild symptoms such as fever, muscle aches, nausea, and diarrhea. However, the invasive form is far more severe and can lead to life-threatening conditions such as septicemia, meningitis, and encephalitis. Pregnancy increases susceptibility to listeriosis as *Listeria* can cross the placental barrier, potentially leading to severe neonatal infections and miscarriage [4]. Newborns, the elderly, and individuals with weakened immune systems are also at high risk. In these populations, listeriosis can cause severe complications, including central nervous system infections and septicemia [5,6]. Listeriosis is recognized as one of the foremost causes of mortality from foodborne diseases within the European Union [7].

The high number of serotypes described for *L. monocytogenes* is evidence of its genetic diversity, which enables a high capacity for adaptation to different environments and conditions [8]. This genetic diversity is reflected in the 13 recognized serotypes, with serotypes 1/2a, 1/2b, 1/2c, and 4b being the most common and associated with most human listeriosis cases. The *L. monocytogenes* adaptation ability complicates its control and eradication efforts.

Although *L. monocytogenes* is generally susceptible to a variety of antibiotics, strains with resistance to multiple antibiotics have been reported. This phenomenon of multidrug resistance has been increasingly observed, and it has been influenced by the widespread and indiscriminate use of antibiotics in agriculture and livestock. Presently, the non-controlled use of antibiotics in feed is prohibited in the European Union and the corresponding regulations are expected to tackle the problems arising from such abusive use [9,10]. While this measure aims to address the increase in multi-resistance to antibiotics, their prior use makes the task an arduous one. The presence of resistance genes highlights the need for continuous surveillance and prudent use of antimicrobials [11,12,13]. Additionally, the horizontal gene transfer of mobile genetic elements, such as plasmids, phage-mediated, and transposons, contributes to the spread of antibiotic resistance among *L.monocytogenes* strains [14]. Also, *L. monocytogenes* possesses a remarkable ability to form biofilms, structures that allow adhesion to surfaces, and resistance to the action of disinfectants and other antimicrobial agents [15]. Biofilms are three-dimensional complex structures composed of bacterial cells embedded in a self-produced matrix of an extracellular substance, mainly composed of exopolysaccharides. This matrix provides physical protection and enhances bacterial resistance to environmental stress, including cleaning and disinfection processes. Thus, biofilm-embedded bacteria exhibit higher resistance to bacteriostatic and bactericidal compounds than planktonic ones [16]. Biofilm formation can be influenced by various factors, including temperature, nutrient availability, and surface materials. For instance, biofilms tend to form more readily on stainless steel and plastic surfaces commonly found in food-processing environments [17]. Biofilms represent, therefore, a persistent source of contamination in food-processing plants, as they protect bacteria from adverse conditions and facilitate their survival in the environment.

These unique capabilities of *L. monocytogenes* make it a constant challenge for the food industry. This pathogen can enter food-processing facilities and equipment through several routes, including workers’ clothes, shoes, or hands, contaminated raw ingredients, or packaging materials. Contaminated equipment and tools can harbor *L. monocytogenes*, especially if they are not adequately cleaned and sanitized. Contaminated water used in processing or cleaning can be a source, too [18]. This persistence of *L. monocytogenes* in food-processing plants is a significant concern because it can lead to continuous contamination of food products. Consequently, the detection and control of *L. monocytogenes* in food-processing equipment and environments are crucial to ensuring food safety and protecting public health. Effective control measures include regular and thorough cleaning and sanitation protocols, the use of antimicrobial agents, and the implementation of strict hygiene practices among personnel. Additionally, advanced detection methods, such as molecular techniques and rapid testing kits, are essential for early identification and control of *L. monocytogenes* contamination [19,20,21].

In this work, we analyze the distribution of various strains belonging to the species under study in terms of genetic diversity, antibiotic resistance, and biofilm generation capacity. These strains were isolated from meat product manufacturing companies in different locations in Spain. Then, we compare them with data from the literature regarding strains isolated in other geographical locations. This comparative analysis aims to identify patterns and differences that could inform better control strategies and improve food-safety practices.

## 2. Materials and Methods

### 2.1. Sampling Procedure

A total of five food-processing plants (FPP) located in Spain (2 in the Basque Country and one each in the Valencian Community, Catalonia, and Andalusia) were sampled for *Listeria* spp. from January to June 2024. A total of 110 samples were collected, 88 from the plants located in the Basque Country and 22 from the rest of the Autonomous Communities. Samples were taken from raw materials (pork, bovine, and poultry), processing equipment, surfaces, and sewage. Samples were collected as follows: Raw materials (50–100 g) were collected using sterile spatulas or tongs and were dropped in sterile 150 mL polypropylene flasks (Deltalab, Barcelona, Spain). Processing equipment and surface samples were collected from 25 cm^2^ using sterile swabs that were kept in 10 mL buffered peptone water tubes (BPW, Oxoid, Hampshire, UK). A swab moistened with BPW was used to rub the surface to be tested with repeated movements in different directions for 2–4 min. The total tested area comprised a square of 5 × 5 cm, and the material of the surfaces was mostly stainless steel, although Teflon-type plastic surfaces were also sampled. Sewage samples (100 mL) were collected and kept in sterile 150 mL polypropylene flasks. To avoid possible alterations, samples were kept at 4 °C until processed in the laboratory, within a maximum time frame of 24 h after collection. The sample collection in each company was carried out on 2 different days with an interval of 2 months between them. Samples were taken at the same locations to ensure the reproducibility of results.

### 2.2. Isolation and Characterization of Strains

Twenty-five grams of raw material samples were weighed and diluted 1:9 (*w*:*w*) with half Fraser broth (Scharlau, Barcelona, Spain) in sterile bags (Seward Ltd., West Sussex, UK). Samples were homogenized in a Stomacher 400 (IUL, Barcelona, Spain) for three minutes. Sewage samples were processed in the same way. Tubes containing processing equipment and surface samples were homogenized by shaking, and BPW was poured into sterile bags containing 90 mL of half Fraser broth and homogenized again.

From this point on, the ISO 11290-1 reference method for *L. monocytogenes* detection [22] was followed. For discarding non-*Listeria monocytogenes* strains, the real-time PCR method iQ-Check™ *Listeria monocytogenes* II kit (Bio-Rad, Hercules, CA, USA) was used [23], following the standard protocol in a CFX 96Touch real-time PCR Detection System (Bio-Rad, Hercules, CA, USA). For the confirmation part, the ALOA^®^ ONE DAY method [24] was used. Greenish colonies with opaque halo were typical of *L. monocytogenes* presumptive strains on ALOA medium (BioMérieux, Marcy-L’Etoile, France). PCR-positive strains that were presumptive on ALOA were classified as *L. monocytogenes*. Greenish colonies without opaque halo were considered presumptive *Listeria* spp. strains. All strains were replated and isolated into a new plate of ALOA medium and kept refrigerated for subsequent identification.

To determine the genus and species of each previously isolated strain, two methodologies were used: API^®^LISTERIA (BioMérieux) biochemical gallery and 16S rDNA PCR. API^®^LISTERIA was used following the manufacturer’s instructions [25]. The 16S rDNA genes were amplified by conventional PCR, following the method described by Chen et al. [26]. DNA from each strain was extracted from two individual colonies that were picked from ALOA medium and suspended in 20 µL of sterile water. Direct DNA extraction was performed by heating samples for 4 min in a microwave at 800 W. The primers *27Fc* [27] and *PUBr* [28] were used (Isogen, Utrecht, The Netherlands). PCR was performed using the NZYTaq II 2× Green Master Mix kit (NZYTech, Lisbon, Portugal) in a QuantStudio^TM^ 5 Real-Time PCR System thermocycler (Applied Biosystems, San Francisco, CA, USA). PCR products were run on a 0.8% agarose gel with 1× TAE (40 mM Tris-acetate-EDTA buffer, pH 8) buffer and photographed under UV light using a BioDoc-It^TM^ Imaging System (Bio-Rad, Marnes La Coquette, France). 16S rDNA PCR products were purified with a GFX PCR DNA Gel Band Purification kit (Cytiva, Little Chalfont, UK). 16S rDNA Sanger sequencing was performed, and DNA homology searches were carried out in the GenBank database using the Basic Local Alignment Search Tool, BLASTN 2.16.1+ [29]. Strains were considered *L. monocytogenes* when coincident profiles with confidence levels higher than 95% were obtained in both methods. Strains were considered *Listeria innocua* or *Listeria welshimeri* using the same criteria. Finally, strains were transferred to cryovials and stored in ultra-freezers at −80 °C available for further testing.

### 2.3. Serogroup Determination

*Listeria monocytogenes* serovar determination was carried out by a modification of the method of Doumith et al. [30]. Three bacterial colonies grown overnight on Columbia agar plates (Bio-Rad, Marnes de la Coquette, France) were emulsified in 50 μL of a 0.25% sodium dodecyl sulfate in 50 mM NaOH solution and incubated at 99 °C for 15 min. A volume of 100 µL of distilled sterile water was added to the mixture and then centrifuged at 11,000× *g* for 10 min. Seventy microliters of the supernatant were collected, and 2 μL was used as a template for the PCR. Amplification reactions were performed in 200 µL tubes (Deltalab) in a final volume of 25 μL. A master mix from the QIAQEN^®^ Multiplex PCR kit (Qiagen GmbH, Hilden, Germany) was used. The five primer sets were added at the following final concentrations: 1.5 μM for *lmo1118*, 1 μM for *ORF2110,* and 0.2 μM for *prs*, *lmo0737*, and *ORF2819*. PCR was performed with an initial denaturation step at 94 °C for 3 min; 35 cycles of 94 °C for 0.40 min, 53 °C for 1.15 min, and 72 °C for 1.15 min; and one final cycle of 72 °C for 7 min in a QuantStudio^TM^ 5 Real-Time PCR System. Two microliters of the reaction mixture were mixed with 1 μL of loading buffer and separated on a 2% agarose gel in a Tris acetate-EDTA buffer (Sigma-Aldrich^®^, St. Louis, MO, USA). The PCR product was visualized by Gel Red^®^ nucleic acid stain (Biotium, Fremont, CA, USA). All primers used in this work are shown in Table 1.

Lineage was established as described by Bonaventura et al. [31]: Lineage I, clustering serotypes 1/2b, 3c, 3b, and 4b; Lineage II, including serotypes 1/2a, 1/2c, and 3a; and Lineage III, grouping serotypes 4a and 4c. Taking into account that the most abundant serotypes linked to foodborne origin and human listeriosis are 1/2a, 1/2b, 1/2c, and 4b, it was considered that Lineage I includes serogroups IIb and IVb, Lineage II includes IIa and IIc and Lineage III includes serogroup IVa.

### 2.4. Biofilm Formation Assay

The biofilm-forming capacity of *Listeria* strains was evaluated by a modification of the method previously described by Bonsaglia et al. [32]. Briefly, bacterial strains were cultured in Tryptic Soy Broth (TSB, Oxoid, Hampshire, UK) at 30 °C overnight. These cultures were diluted 1:99 (v:v) until a bacterial density of ca. 10^7^ CFU/mL. Aliquots of 200 µL of each bacterial suspension were plated into individual wells of sterile flat bottom 96-well microplates (Nunclon^TM^ Delta surface, Thermo-Scientific, Roskilde, Denmark). TSB was used as a negative control. The microplates were incubated for 48 h, without stirring, at 25 °C and 37 °C. Following incubation, the wells were carefully washed three times with sterile water for planktonic cell removal and dried at 50 °C for 1 h. Biofilms were stained with 200 μL 0.1% crystal violet (CV) for 10 min at room temperature. Then, three washes of 200 µL/well sterile water were done to remove unbound CV, and plates were air-dried at room temperature. Finally, the bound dye was recovered with 200 µL/well of 30% (v:v) acetic acid in water. Absorbance at 600 nm was measured in a microplate reader BioTek Synergy SH1 MG (Agilent, Santa Clara, CA, USA). For the adhesion capacity measure, the procedure of Stepanovic et al. was followed [33]. The cut-off optical density value (OD_C_) for the microtiter-plate test was set as three standard deviations above the mean OD of the negative control. Based upon the optical densities of bacterial biofilms, strains were then classified as non-adherent (OD ≤ OD_C_), weakly adherent (OD_C_ < OD ≤ 2 × OD_C_), moderately adherent (2 × OD_C_ < OD ≤ 4 × OD_C_), and strongly adherent (4 × OD_C_ > OD). All tests were performed five times, and the results were averaged.

### 2.5. Antibiotic Resistance

*Listeria monocytogenes* isolates were subjected to the disk-diffusion method, according to CLSI [34] and EURECAST [35] to identify their resistance to a panel of 23 antibiotics from 14 classes: amoxicillin + clavulanic acid (AMC, ß-lactams, 20/10 µg, EUCAST 2024), ampicillin (AMP, ß-lactams, 10 µg, EUCAST 2024), amoxicillin (AMX, ß-lactams, 25 µg, CLSI 2024 (considered to be penicillin)), chloramphenicol (CHL, anfenicols, 30 µg, CLSI 2024), ciprofloxacin (CIP, lincosamides, 5 µg, EUCAST 2024), clindamycin (CLI, lincosamides, 2 µg, EUCAST 2024), cefotaxime (CTX, cephalosporins, 20 µg, EUCAST 2024), erythromycin (ERY, macrolides, 15 µg, EUCAST 2024), fosfomycin (FOS, phosphonic acid derivatives, 50 µg, EUCAST 2024), fusidic acid (FUS, steroids, 5 µg, EUCAST 2024), gentamicin (GEN, aminogycosides, 10 µg, EUCAST 2024), imipenem (IPM, carbapenems, 10 µg, EUCAST 2024), kanamycin (KAN, aminoglycosides, 30 µg, CLSI 2024), nalidixic acid (NAL, quinolones, 30 µg, EUCAST 2024), oxacylin (OXA, ß-lactams, 1 µg, EUCAST 2024), bencylpenicillin (PCG, ß-lactams, 0.5 µg, EUCAST 2024), penicillin (PEN, ß-lactams, 10 µg, CLSI 2024), rifampicin (RIF, ansamycins, 5 µg, EUCAST 2024), sulfonamide (SUL, sulfonamides, 200 µg, CLSI 2024), trimethoprim-sulfamethoxazole (SXT, sulfonamides, 25 µg, EUCAST 2024), tetracycline (TET, tetracyclines, 30 µg, EUCAST 2024), trimetoprim (TMP, sulfonamides, 5 µg, CLSI 2024) and vancomycin (VAN, glycopeptides, 30 µg, EUCAST 2024). All the antibiotics, except AMP, GEN, KAN, PEN, VAN, and PCG, were purchased as antimicrobial susceptibility test discs from Oxoid (Basingstoke, UK). AMP was from Fisher Bioreagens (Geel, Belgium), GEN and VAN were from Sigma-Aldrich (St. Louis, MO, USA), KAN was from FLUKA-Sigma (Buchs, Switzerland), PEN was from Merck (Darmstadt, Germany) and PCG was from Selleck Chemicals (Houston, TX, USA). These antibiotics were prepared into a Class 2 Biosafety Cabinet and weighed aseptically according to the indications above, dissolved in 20 µL sterile distilled water, and poured onto cellulose discs. They were kept at room temperature until dried. Once prepared, discs were kept at 4 °C for no more than 24 h until use.

Briefly, the disk-diffusion method was performed in Mueller-Hinton Agar (Sigma-Aldrich, Darmstadt, Germany). Each inoculum was prepared as an individual overnight culture (~10^8^ CFU/mL) in Mueller-Hinton broth (Himedia, Nashik, India) at 37 °C. The microbial suspensions were individually spread with a sterile swab over the entire agar surface in three directions, and the corresponding antibiotic disks were subsequently applied (with a maximum of 4 disks per plate). Plates were incubated at 37 °C for 24 h. After incubation, the inhibition zones were measured in mm with a ruler and further interpreted.

*Staphylococcus aureus* ATCC 25923 was used as a control. Antimicrobial resistance interpretation was based on CLSI and EUREST recommendations, and they were determined using as a first option the cut-off values established for *L. monocytogenes* (AMP, ERY, SXT, PCG, CTX, NAL). When *L. monocytogenes* was not considered to be a model for any of the antibiotics, the chosen models were *Staphylococcus* sp. (CIP, CLI, CHL, GEN, KAN, OXA, PEN, RIF, TET, FUS, FOS, SUL, TMP), *Enterococcus* sp. (VAN), *Bacillus* sp. (IMP) and *Burkholderia pseudomallei* (AMC). Strains were classified as presenting resistance (R), intermediate resistance (I), or susceptibility (S) to the tested antibiotic.

### 2.6. Miscellaneous

All chemicals were, at least, of analytical grade and purchased from Fisher Scientific or Scharlau. The chi-square test was used to verify the homogeneity in the distribution of the categorical variables analyzed in this work. Differences were considered significant at *p* < 0.05.

## 3. Results and Discussion

Bacterial biofilm-forming capacity analysis allows for assessing and taking appropriate measures regarding the hygienical and sanitary status of food-processing facilities. Observations indicated that this capability was extensively distributed among the *Listeria* spp. present in the facilities under study. Genetic characterization of *L. monocytogenes* allows for the monitoring of strain lineages present in food-processing facilities. It also enables traceability and response to potential foodborne outbreaks. The results of this study showed that antimicrobial resistance is a widespread phenomenon. A review of the relevant literature suggests the need to work toward unifying criteria for analyzing the evolution of this phenomenon over time and across different geographic locations.

### 3.1. Listeria spp. Implantation in Food-Processing Plants

Table 2 shows the results of the genetic characterization and the biofilm-forming capacity of the *Listeria* isolates obtained after the sampling in the food-processing plants under study. Detailed information about the biofilm-forming capacity of each strain at the different conditions studied can be checked in the Supplementary Material File (Tables S1–S4). Table 3 and Table 4 summarize the results concerning species distribution.

Notably, *Listeria monocytogenes* was isolated in all the food-processing plants, while *L. innocua* and/or *L. welshimeri* were found in four out of the five plants (exception: FPP5). The analysis of samples from the Basque Country showed the following results. When *Listeria* isolates were categorized by production plant and origin (either food or environmental), the species distribution exhibited *p*-values of 0.442 and 0.177, respectively (Table 3). These values indicate that the differences were not statistically significant. Similarly, when isolates from both environmental and food origins were combined and compared across the food-processing plants, the species distribution yielded a *p*-value of 0.489, suggesting no significant differences between them. Furthermore, the species distribution did not show significant variations between the two food-processing plants located in the Basque Country.

As the number of observations per species was not high enough to ensure adequate statistical results, the *Listeria* isolates from the plants from the Valencian Community, Catalonia, and Andalusia were grouped, and the species distribution between the isolates from the Basque Country and the rest of Spain was analyzed in Table 4. The species distribution yielded a *p*-value of 0.022, demonstrating significant differences in the distribution of these species between clusters.

As summarized in Table 5, a comprehensive review of the literature indicates that *L. monocytogenes*, *L. innocua*, *L. welshimeri*, *L. ivanovii,* and/or *L. seeligeri* are commonly found within the food chain across several geographical locations [36,37,38,39,40]. The data analysis revealed that the distribution of these species was highly heterogeneous, yielding *p*-values of less than 0.05 in all cases.

This observed heterogeneity could be attributed to several factors, including the diverse environmental conditions, the nature of raw materials used, the processes they undergo, the expertise of the operators, and the specific procedures developed in each plant. These variables play a crucial role in shaping the distribution patterns of *Listeria* species and greatly contribute to the observed heterogeneity. The low number of *L. monocytogenes* strains isolated in the German study may be related to the nature of the samples, as all of them were environmental, collected primarily from vegetable processing plants. While some vegetables provide a suitable environment for the growth of *L. monocytogenes*, the low pH of many fruits hinders the growth of this microorganism. In the Italian case, where pig carcasses were studied, their processing appears to play a crucial role in controlling the presence of *L. monocytogenes*. The entire process, from transporting the animals from the farm to the slaughterhouse to cutting pig carcasses, paying special attention to the processing of heads, which appears to be the main source of contamination, is carried out with the aim of minimizing it, given the high added value of this meat, especially when it is used to manufacture hams. In South African works and this study, there was no presence of either *L. ivanovii* or *L. seeligeri*, perhaps due to the different prevalence of these microorganisms in the samples under study or due to geographical distribution issues. Integrating this knowledge into the process control system of a food-processing plant is essential for the effective management of food-safety risks. By understanding the factors that influence the distribution of *Listeria* species, food industry professionals could implement more targeted and effective control measures.

### 3.2. Listeria monocytogenes Serogroups

The distribution of the observed *L. monocytogenes* serogroups is shown in Table 6. Due to the limited number of observations per species and food-processing plant, ensuring robust statistical results was challenging. Consequently, the samples were clustered based on their geographical origins. This approach revealed a *p*-value of 0.006, indicating significant differences in the distribution of serogroups between the strains isolated in the Basque Country and those from the rest of Spain. The most frequently identified serogroup of isolates from Basque Country was IIb (44% of total isolates), whereas the most prevalent serogroup of isolates from the rest of Spain was IIa (50% of total isolates). Ninety-six percent (24/25) of the strains isolated in the two food-processing plants from the Basque Country and 100% (12/12) of those found in the ones from the rest of Spain were clustered into serogroups IIa, IIb, IIc, or IVb, which correspond to serotypes 1/2a, 1/2b, 1/2c, and 4b, respectively.

These serotypes are most commonly linked to human listeriosis cases [41]. It is a matter of concern that almost all the *L. monocytogenes* isolates belong to serogroups with the highest incidence of listeriosis in humans. However, it is reassuring to know that the food-processing methodologies and the hygienic and sanitary measures implemented in the food-processing plants under study are sufficiently robust to ensure the absence of this pathogen in their food-safety controls, guaranteeing, therefore, the commercialization of safe products for consumers.

As illustrated in Table 7, a review of the literature reveals that the serogroup distribution does not show significant differences between the analyzed studies [11,12,13,38,42,43,44,45,46]. However, data analysis does not demonstrate a clear coincidence between the distribution of the serogroups and their source of isolation. Therefore, no clear relation between the examined parameters can be established. The most frequently identified serogroup of isolates from Basque Country under the present study was IIb (44%), whereas the most prevalent serogroup of isolates from the rest of the studies were IIa (44–77%) [11,12,13,38,42,43,45,46] and IIc (69%) [44].

Although not commonly associated with food, some strains belonging to serogroup IVa, also observed in this study, have already been isolated from food and food production facilities. Henriques et al. [47] found, during a series of audits in pork, veal, and poultry meat-processing plants from Portugal, 18 strains out of a total of 62 belonging to this serogroup. Previously, Wu et al. [48] also found, from an extensive sampling of retailed raw food samples from China, including vegetables, mushrooms, meat, meat products, and fish products, 14 strains out of 248 belonging to the serogroup. The serogroup distribution of *L. monocytogenes* can vary significantly depending on various factors, including geographical location, environmental conditions, and the type of food matrices involved, but there is no consistent pattern that links serogroup distribution directly to the type of food matrix [49]. The analysis of the literature shows that, in any case, the prevalent serogroups in food and food-processing environments are serogroups IIa, IIb, IIc, and IVb, which correspond to serotypes 1/2a, 1/2b, 1/2c, and 4b, respectively, the most commonly linked to human listeriosis cases.

### 3.3. Biofilm Formation Capacity

The biofilm-forming capacity of *Listeria* isolates was analyzed at two temperatures: 25 °C and 37 °C. It is well established that *Listeria* species can grow at temperatures ranging from 0 °C to 45 °C, with optimal growth occurring between 30 °C and 37 °C. In the food industry, food handling and processing steps are typically carried out at controlled temperatures. However, temperature control could be less stringent at certain moments, making it crucial to understand the behavior of these species under varying conditions.

The results, presented in Table 2, indicate that after 48 h at 25 °C only two out of 37 (5.4%) strains of *L. monocytogenes* were unable to form biofilms, two strains exhibited moderate biofilm production, while the remaining 33 strains demonstrated strong biofilm production. Notably, none of the 19 *L. innocua* isolates showed the ability to generate biofilms, and among the 19 *L. welshimeri* strains, only two exhibited a weak capacity for biofilm production.

An increase in growth temperature up to 37 °C had a pronounced effect on the biofilm formation capacity of these microorganisms. Among the 33 *L. monocytogenes* strains that produced strong biofilms at 25 °C, 17 reverted to moderate biofilm producers, while the remaining 15 strains maintained as strong producers. Conversely, the two strains that exhibited moderate biofilm production at 25 °C remained as moderate biofilm producers at 37 °C. Finally, among the two non-biofilm producers at 25 °C, one reverted to a weak producer while the other remained as a non-producer.

This behavior is recorded in Table 8, where the strains are also grouped according to their lineage. Individual strain information can be checked in the Supplementary Material File (Tables S1–S4). As described by Di Bonaventura et al. [31], no statistically significant differences were observed between lineages (*p* < 0.05), regardless of the growth temperature and the distribution of cases concerning the biofilm production capacity. The distribution of profiles of variation in the intensity of biofilm formation between 25 °C and 37 °C also showed no significant differences between lineages (Table 8 ^d^). It is well known that serotypes are characterized by the different properties of the bacterial surface, which largely depend on their components and topological characteristics, such as the presence of fimbriae and flagella [49,50]. These characteristics can be closely related to the ability of different lineages/serogroups/serotypes to form biofilms and how they respond to changes in growth temperature.

The behavior of the other *Listeria* species also showed a different pattern at 37 °C. Specifically, four, eight, and seven *L. innocua* strains formed weak, moderate, and strong biofilms, respectively. Among the 17 non-biofilm producers *L. welshimeri* strains at 25 °C, four remained non-biofilm-forming at 37 °C, whereas six and seven strains converted to weak and moderate biofilm producers, respectively. The two strains that were weak biofilm-forming at 25 °C converted to moderate biofilm-forming at 37 °C. *Listeria* species have a versatile metabolism that enables them to thrive in adverse environmental conditions, such as low temperatures, high salinity, and a broad pH range. This adaptability allows them to survive and persist in various settings, including food-processing environments. One of the key survival strategies of *Listeria* spp. is their ability to form biofilms. These biofilms significantly enhance their potential to establish themselves on food-contact surfaces and equipment within food-processing plants, where they can persist for extended periods.

Once established, biofilms become a major source of cross-contamination, facilitating the spread of *Listeria* spp. throughout the environment. This can lead to widespread colonization, posing a serious risk to food safety. Moreover, biofilms provide additional protection to the microorganisms by acting as a physical barrier that shields them from the action of disinfectants and other cleaning agents. This protective layer makes it challenging to eradicate *Listeria* from contaminated surfaces, necessitating rigorous and continuous sanitation efforts.

### 3.4. Antibiotic Resistance

As shown in Table 9, the profiles for antimicrobial resistance (AMR) of all the *L. monocytogenes* strains isolated in this study were determined against a panel of 23 antibiotics from 14 classes. Table 10 summarizes the AMR profiles observed in this study and those recovered from the literature [11,12,13,36,43]. The results indicate a wide distribution of AMR among *L. monocytogenes*. All strains from this study showed AMR to at least six different antibiotics from five different families (AMC^I^, CIP^I^, CTX^R^, FUS^R^, NAL^R^, and OXA^R^), 35 showed AMR to this profile plus CLI^R^, and 34 added FOS^R^ to the previous seven antibiotics.

The AMR profiles found in this study greatly differ from those observed in the literature. It is noteworthy that the studies analyzed from the literature do not use a unified criterion for selecting the antibiotics to be analyzed. Thus, the results found in the literature are partial, as each study does not include a representative sample of available antibiotics.

In this study, different antibiotics from different families were analyzed in order to obtain an overview of the scope of AMR and their impact on the development of resistance to different antibiotic families. These observations are summarized in Table 11. Quinolone (NAL) resistance was only verified in one study from the literature analyzed, and as expected, 100% of the strains studied were resistant. The resistance of *L. monocytogenes* to NAL is well known. NAL is, therefore, used in the composition of culture media for the selective isolation of *L. monocytogenes* [1]. Cephalosporins, phosphonic acid derivatives, sulfonamides, and ß-lactams followed in the ranking of antibiotic resistance.

The presence of different AMR profiles poses a risk of transmission of these resistances between strains. In our highly interconnected and globalized world, it is highly reasonable that raw materials sourced from distant regions can carry strains with local resistance to various destinations. When native bacterial strains encounter these foreign strains, the exchange of resistance genes becomes feasible. This interaction not only facilitates the spread of existing resistances but also contributes to the increase and diversification of AMR profiles. Consequently, this dynamic can lead to more complex and widespread resistance patterns, posing a substantial challenge to public health and the effectiveness of current antimicrobial treatments.

The transfer of genetic material through plasmids by conjugation is a well-documented phenomenon. Plasmids can serve as shuttles for the spread of AMR genes. Such cases have already been described. Transmission of a resistance gene against TET via a small plasmid has already been reported [51], and the conjugative transfer of four resistance genes against CHL, ERY, TET, and streptomycin by a single plasmid has also been observed [52]. Moreover, horizontal transfer between different species and genera has also been described [53].

The potential role of temperate bacteriophages and transposons as vectors for the transfer of antibiotic resistance genes should not be overlooked. Their involvement in the transfer of biocide tolerance genes has already been described [54]. These mechanisms provide degrees of freedom to the transmission of ARMs and force the food industry to maintain and continually improve the high standards required in the procedures aimed at ensuring food safety.

## 4. Conclusions

*Listeria* spp. is widely distributed in nature and can thrive in various environments under a broad range of physical and chemical parameters. Their presence in raw material production and food-processing environments is common. Exhaustive control of working conditions and hygiene measures are required to ensure food safety and to avoid public-health problems in the food chain arising from the uncontrolled entry of foodborne *Listeria monocytogenes*.

The *Listeria* species distribution did not show significant variations between the two food-processing plants located in the Basque Country. Significant differences in species distribution were observed between samples from the Basque Country and the cluster formed by the food-processing plants from the Valencian Community, Catalonia, and Andalusia.

*Listeria* species can form biofilms, which enhance their ability to persist on surfaces and equipment in food-processing plants. Biofilm formation varies with temperature, with stronger biofilm formation observed at 37 °C compared to 25 °C.

Significant differences in the distribution of *L. monocytogenes* serogroups were found between strains isolated in the Basque Country and those from the rest of Spain. The majority of strains belonged to serogroups usually found in human listeriosis cases.

*Listeria monocytogenes* strains exhibited diverse antibiotic multi-resistance profiles, posing a risk of horizontal transmission between strains and increasing the risk for food safety and public health.

## Figures and Tables

**Table 1:** PCR primers used in this study.

Primers	Length	Sequence 5′→3′	Source
*27FC* (F)	18	AGT TTG ATC CTG GCT CAG	[26]
*PUBR* (R)	19	CCC GGG AAC GTA TTC ACC G	[27]
*lmo0737* (F)	20	AGG GCT TCA AGG ACT TAC CC	[29]
*lmo0737* (R)	20	ACG ATT TCT GCT TGC CAT TC	[29]
*lmo1118* (F)	20	AGG GGT CTT AAA TCC TGG AA	[29]
*lmo1118* (R)	20	CGG CTT GTT CGG CAT ACT TA	[29]
*ORF2819* (F)	20	AGC AAA ATG CCA AAA CTC GT	[29]
*ORF2819* (R)	21	CAT CAC TAA AGC CTC CCA TTG	[29]
*ORF2110* (F)	21	AGT GGA CAA TTG ATT GGT GAA	[29]
*ORF2110* (R)	21	CAT CCA TCC CTT ACT TTG GAC	[29]
*prs* (F)	22	GCT GAA GAG ATT GCG AAA GAA G	[29]
*prs* (R)	22	CAA AGA AAC CTT GGA TTT GCG G	[29]

**Table 2:** Genetic and biofilm-forming capacity characterization of the *Listeria* strains isolated from the food-processing plants under study. LMO: *L. monocytogenes*; LIN: *L. innocua*; LWE: *L. welshimeri;* FPP1 and FPP2: food-processing plants from the Basque Country; FPP3: food-processing plant from the Valencian Community; FPP4: food-processing plant from Catalonia; and FPP5: food-processing plant from Andalusia. FPE: food-processing equipment; and N.A.: not assayed.

				Genetic Characterization		Biofilm-Forming Capacity	
Strain	Origin	Geographical Origin	Source	Serogroup	Lineage	25 °C	37 °C
LMO391	FPP1	Basque Country	Pork/Food	IIb	I	Strong	Moderate
LMO392	FPP1	Basque Country	Pork/Food	IVb	I	Strong	Moderate
LMO393	FPP1	Basque Country	Beef/Food	IIa	II	Strong	Moderate
LMO394	FPP1	Basque Country	Beef/Food	IIb	I	Strong	Moderate
LMO395	FPP1	Basque Country	Sewage/Drain/Environmental	IVb	I	Strong	Moderate
LMO396	FPP1	Basque Country	Sewage/Drain/Environmental	IVb	I	Strong	Moderate
LMO397	FPP1	Basque Country	Sewage/Drain/Environmental	IIb	I	Strong	Strong
LMO398	FPP2	Basque Country	FPE/Environmental	IIb	I	Strong	Strong
LMO399	FPP2	Basque Country	Sewage/Drain/Environmental	IIa	II	Strong	Moderate
LMO400	FPP2	Basque Country	Sewage/Drain/Environmental	IIb	I	Non-Biofilm	Non-Biofilm
LMO401	FPP2	Basque Country	FPE/Environmental	IIb	I	Strong	Strong
LMO402	FPP2	Basque Country	Sewage/Drain/Environmental	IIc	II	Strong	Moderate
LMO403	FPP2	Basque Country	FPE/Environmental	IIb	I	Strong	Strong
LMO404	FPP2	Basque Country	FPE/Environmental	IIb	I	Strong	Strong
LMO405	FPP2	Basque Country	Pork/Food	IIa	II	Strong	Strong
LMO406	FPP5	Andalusia	FPE/Environmental	IIc	II	Strong	Strong
LMO407	FPP4	Catalonia	Poultry/Food	IIa	II	Strong	Weak
LMO408	FPP4	Catalonia	Poultry/Food	IIa	II	Strong	Strong
LMO409	FPP4	Catalonia	Poultry/Food	IIa	II	Strong	Strong
LMO410	FPP4	Catalonia	Poultry/Food	IIa	II	Moderate	Moderate
LMO411	FPP4	Catalonia	Poultry/Food	IIc	II	Strong	Moderate
LMO412	FPP4	Catalonia	Poultry/Food	IIc	II	Strong	Strong
LMO413	FPP4	Catalonia	Poultry/Food	IIa	II	Strong	Strong
LMO414	FPP4	Catalonia	Poultry/Food	IIa	II	Strong	Moderate
LMO415	FPP1	Basque Country	Pork/Food	IVa	III	Strong	Strong
LMO416	FPP1	Basque Country	Pork/Food	IVb	I	Strong	Moderate
LMO417	FPP1	Basque Country	Beef/Food	IIa	II	Strong	Moderate
LMO418	FPP1	Basque Country	FPE/Environmental	IIb	I	Strong	Strong
LMO419	FPP1	Basque Country	Sewage/Drain/Environmental	IIa	II	Strong	Strong
LMO420	FPP1	Basque Country	Sewage/Drain/Environmental	IVb	I	Strong	Moderate
LMO421	FPP1	Basque Country	Sewage/Drain/Environmental	IIb	I	Strong	Moderate
LMO422	FPP2	Basque Country	FPE/Environmental	IIb	I	Strong	Strong
LMO423	FPP2	Basque Country	Floor/Environmental	IIc	II	Strong	Moderate
LMO424	FPP2	Basque Country	Sewage/Drain/Environmental	IIc	II	Strong	Moderate
LMO425	FPP3	Valencian Community	Poultry/Food	IVb	I	Non-Biofilm	Weak
LMO426	FPP3	Valencian Community	Poultry/Food	IVa	III	Moderate	Moderate
LMO428	FPP5	Andalusia	Pork/Food	IIc	II	Strong	Moderate
LIN014	FPP1	Basque Country	Pork/Food	N.A.	N.A.	Non-Biofilm	Strong
LIN015	FPP1	Basque Country	Pork/Food	N.A.	N.A.	Non-Biofilm	Strong
LIN016	FPP1	Basque Country	Beef/Food	N.A.	N.A.	Non-Biofilm	Moderate
LIN017	FPP1	Basque Country	Sewage/Drain/Environmental	N.A.	N.A.	Non-Biofilm	Moderate
LIN018	FPP1	Basque Country	Sewage/Drain/Environmental	N.A.	N.A.	Non-Biofilm	Moderate
LIN019	FPP1	Basque Country	Sewage/Drain/Environmental	N.A.	N.A.	Non-Biofilm	Strong
LIN020	FPP2	Basque Country	Sewage/Drain/Environmental	N.A.	N.A.	Non-Biofilm	Moderate
LIN021	FPP2	Basque Country	FPE/Environmental	N.A.	N.A.	Non-Biofilm	Moderate
LIN022	FPP2	Basque Country	FPE/Environmental	N.A.	N.A.	Non-Biofilm	Moderate
LIN023	FPP2	Basque Country	Poultry/Food	N.A.	N.A.	Non-Biofilm	Moderate
LIN024	FPP2	Basque Country	Poultry/Food	N.A.	N.A.	Non-Biofilm	Strong
LIN025	FPP2	Basque Country	Pork/Food	N.A.	N.A.	Non-Biofilm	Strong
LIN026	FPP1	Basque Country	Beef/Food	N.A.	N.A.	Non-Biofilm	Weak
LIN027	FPP1	Basque Country	Pork/Food	N.A.	N.A.	Non-Biofilm	Weak
LIN028	FPP1	Basque Country	Sewage/Drain/Environmental	N.A.	N.A.	Non-Biofilm	Strong
LIN029	FPP1	Basque Country	FPE/Environmental	N.A.	N.A.	Non-Biofilm	Strong
LIN030	FPP1	Basque Country	FPE/Environmental	N.A.	N.A.	Non-Biofilm	Weak
LIN031	FPP1	Basque Country	Pork/Food	N.A.	N.A.	Non-Biofilm	Moderate
LIN032	FPP3	Valencian Community	Poultry/Environmental	N.A.	N.A.	Non-Biofilm	Weak
LWE002	FPP1	Basque Country	Sewage/Drain/Environmental	N.A.	N.A.	Non-Biofilm	Weak
LWE003	FPP2	Basque Country	Floor/Environmental	N.A.	N.A.	Non-Biofilm	Weak
LWE005	FPP2	Basque Country	Sewage/Drain/Environmental	N.A.	N.A.	Non-Biofilm	Weak
LWE006	FPP2	Basque Country	Sewage/Drain/Environmental	N.A.	N.A.	Non-Biofilm	Weak
LWE007	FPP4	Catalonia	Poultry/Food	N.A.	N.A.	Non-Biofilm	Non-Biofilm
LWE008	FPP4	Catalonia	Poultry/Food	N.A.	N.A.	Non-Biofilm	Weak
LWE009	FPP4	Catalonia	Poultry/Food	N.A.	N.A.	Non-Biofilm	Non-Biofilm
LWE010	FPP4	Catalonia	Poultry/Food	N.A.	N.A.	Non-Biofilm	Non-Biofilm
LWE011	FPP4	Catalonia	Poultry/Food	N.A.	N.A.	Non-Biofilm	Non-Biofilm
LWE012	FPP4	Catalonia	Poultry/Food	N.A.	N.A.	Non-Biofilm	Weak
LWE013	FPP1	Basque Country	Pork/Food	N.A.	N.A.	Non-Biofilm	Moderate
LWE014	FPP1	Basque Country	Sewage/Drain/Environmental	N.A.	N.A.	Weak	Moderate
LWE015	FPP2	Basque Country	Floor/Environmental	N.A.	N.A.	Non-Biofilm	Moderate
LWE016	FPP2	Basque Country	Sewage/Drain/Environmental	N.A.	N.A.	Non-Biofilm	Moderate
LWE017	FPP2	Basque Country	Sewage/Drain/Environmental	N.A.	N.A.	Non-Biofilm	Moderate
LWE018	FPP2	Basque Country	Poultry/Food	N.A.	N.A.	Non-Biofilm	Moderate
LWE019	FPP2	Basque Country	Poultry/Food	N.A.	N.A.	Non-Biofilm	Moderate
LWE020	FPP3	Valencian Community	Poultry/Food	N.A.	N.A.	Non-Biofilm	Moderate
LWE021	FPP3	Valencian Community	Poultry/Food	N.A.	N.A.	Weak	Moderate

**Table 3:** Species distribution according to their geographical origin and source of isolation. FPP1 and FPP2: food-processing plants from the Basque Country; FPP3: food-processing plant from the Valencian Community; FPP4: food-processing plant from Catalonia; and FPP5: food-processing plant from Andalusia. FPE: food-processing equipment; Sew.: sewage; and /: not sampled. *p*-values from Χ^2^ analysis are shown.

			Environmental				Food			
			FPE	Floor	Sew.	Total	Beef	Pork	Poultry	Total
FPP1	*L. monocytogenes*		1	0	6	7	3	4	/	7
	*L. innocua*		1	0	4	5	2	3	/	5
	*L. welshimeri*		0	0	2	2	0	1	/	1
FPP2	*L. monocytogenes*		5	1	4	10	0	1	0	1
	*L. innocua*		3	0	0	3	1	2	2	5
	*L. welshimeri*		0	2	4	6	0	0	2	2
FPP3	*L. monocytogenes*		/	/	/	/	/	/	2	2
	*L. innocua*		/	/	/	/	/	/	1	1
	*L. welshimeri*		/	/	/	/	/	/	2	2
FPP4	*L. monocytogenes*		/	/	/	/	8	0	6	14
	*L. innocua*		/	/	/	/	/	/	/	/
	*L. welshimeri*		/	/	/	/	/	/	/	/
FPP5	*L. monocytogenes*		1	0	0	1	/	1	/	1
	*L. innocua*		/	/	/	/	/	0	/	0
	*L. welshimeri*		/	/	/	/	/	0	/	0
	**Environmental**		**Food**				**Environmental + Food**			
Strains	**FPP1**	**FPP2**	**FPP1**		**FPP2**		**FPP1**		**FPP2**	
*L. monocytogenes*	7	10	7		1		14		11	
*L. innocua*	5	4	5		4		10		8	
*L. Welshimeri*	2	6	1		2		3		8	
Χ^2^ analysis (*p*-values)	0.442		0.177				0.489			

**Table 4:** Summary of species distribution according to their gathered geographical origin and kind of isolation sample. FPP1 + FPP2: food-processing plants from the Basque Country; FPP3 + FPP4 + FPP5: food-processing plants from the Valencian Community, Catalonia, and Andalusia; and /: not sampled. *p*-value from Χ^2^ analysis is shown.

		Environmental	Food	Total
FPP1 + FPP2	*L. monocytogenes*	17	8	25
	*L. innocua*	8	10	18
	*L. welshimeri*	8	3	11
FPP3 + FPP4 + FPP5	*L. monocytogenes*	1	11	12
	*L. innocua*	/	1	1
	*L. welshimeri*	/	8	8
*p*-value (FPP1 + FPP2) vs. (FPP3 + FPP4 + FPP5)				0.022

**Table 5:** *Listeria* spp. distribution profiles (number of strains isolated from each species) found in this study and literature. The geographical origin of each reference is shown. Values from the statistical analysis using chi-square are shown.

**Species/Reference**	This Study, Basque Country	South Africa [35]	Germany [36]	Mid-East and South-East Spain [37]	South Africa [38]	Italy [39]
*L. monocytogenes*	25	37	7	132	3	3
*L. innocua*	18	65	8	89	38	130
*L. welshimeri*	11	10	0	0	3	28
*L. ivanovii*	0	0	5	5	0	17
*L. seeligeri*	0	0	19	6	0	0
	**Χ^2^ analysis (*p*-values)**					
This study (Basque Country, Spain)						
South Africa [36]	5.97·10^−8^					
Germany [37]	8.92·10^−15^	1.40·10^−8^				
Mid-east/south-east Spain [38]	1.22·10^−9^	3.15·10^−8^	1.62·10^−16^			
South Africa [39]	7.25·10^−7^	0.00195	6.69·10^−9^	6.26·10^−11^		
Italy [40]	2.51·10^−17^	9.06·10^−14^	2.48·10^−22^	5.94·10^−35^	0.016	

**Table 6:** Serogroup distribution of the different *L. monocytogenes* isolates from food-processing plants under study. Table shows the number and percentage of strains belonging to each serogroup isolated from food-processing plants in the Basque Country and from the rest of Spain. FPP1 and FPP2: food-processing plants from the Basque Country; FPP3: food-processing plant from the Valencian Community; FPP4: food-processing plant from Catalonia; and FPP5: food-processing plant from Andalusia.

Serogroup	FPP1	FPP2	FPP1 + FPP2	FPP3 + FPP4 + FPP5
IIa	3 (21%)	2 (18%)	5 (20%)	6 (50%)
IIb	5 (36%)	6 (55%)	11 (44%)	0 (0%)
IIc	0 (0%)	3 (27%)	3 (12%)	4 (33%)
IVa	1 (7%)	0 (0%)	1 (4%)	0 (0%)
IVb	5 (36%)	0 (0%)	5 (20%)	2 (17%)
**Total**	14 (100%)	11 (100%)	25 (100%)	12 (100%)

**Table 7:** Serogroup distribution of different *L. monocytogenes* isolates from food-processing plants in this study and literature. Table shows the number and percentage of strains belonging to each serogroup and human-listeriosis-related serogroups. The geographical origin and the type of food matrices analyzed in each reference are shown. Serogroup distributions were analyzed using the chi-square statistical test, and *p*-values from the analysis are shown. Values of *p* > 0.05 (underlined and bold) indicate no significant differences between studies.

Reference/Serogroup			IIa	IIb	IIc	IVa	IVb	IIa + IIb + IIc + IVb		
This study (Meat/Basque Country, Spain)			5 (20%)	11 (44%)	3 (12%)	1 (4%)	5 (20%)	24 (96%)		
Pork/Spain [42]			240 (56%)	63 (15%)	105 (24%)	0 (0%)	23 (5%)	431 (100%)		
Pork/Spain [43]			43 (74%)	7 (12%)	7 (12%)	0 (0%)	1 (2%)	58 (100%)		
Meat/China [13]			55 (45%)	24 (19%)	35 (29%)	0 (0%)	9 (7%)	123 (100%)		
Poultry/Castilla y Leon, Spain [44]			39 (27%)	2 (1%)	102 (69%)	0 (0%)	4 (3%)	147 (100%)		
Meat/Poland [45]			36 (51%)	10 (15%)	15 (21%)	0 (0%)	9 (13%)	70 (100%)		
Meat, Dairy/Romania [12]			7 (44%)	3 (19%)	2 (12%)	0 (0%)	4 (25%)	16 (100%)		
Dairy/Castilla y Leon, Spain [46]			33 (72%)	6 (13%)	2 (4%)	0 (0%)	5 (11%)	46 (100%)		
Meat, dairy, fish, vegetables/France [11]			68 (77%)	9 (10%)	11 (12%)	0 (0%)	1 (1%)	89 (100%)		
Meat, fish, vegetables/Spain [38]			24 (69%)	2 (6%)	6 (17%)	0 (0%)	3 (8%)	35 (100%)		
**Χ^2^ Analysis**	**This Study**	[42]	[43]	[13]	[44]	[45]	[12]	[46]	[11]	[38]
This study										
[42]	1.23·10^−8^									
[43]	3.53·10^−5^	0.046								
[13]	0.13·10^−2^	**0.403**	0.011							
[44]	6.90·10^−16^	1.49·10^−21^	5.08·10^−13^	7.61·10^−12^						
[45]	0.5·10^−2^	**0.124**	0.024	**0.288**	4.45·10^−11^					
[12]	**0.197**	0.25·10^−2^	0.001	0.015	2.11·10^−5^	**0.276**				
[46]	0.16·10^−2^	0.91·10^−2^	**0.137**	0.004	1.20·10^−13^	**0.055**	0.035			
[11]	1.5·10^−7^	0.98·10^−2^	**0.246**	0.12·10^−2^	7.28·10^−10^	0.003	0.75·10^−3^	0.51·10^−2^		
[38]	0.95·10^−3^	0.027	0.32·10^−2^	**0.066**	0.12·10^−2^	**0.372**	**0.849**	0.013	0.19·10^−2^	

**Table 8:** Biofilm-forming capacity of *L. monocytogenes* isolates as a function of their lineage. ^a^ Number of strains unable (non-biofilm) and able to produce weak, moderate, and strong biofilms after 48 h at 25 °C or 37 °C. ^b^ *p*-values correlating Lineages at 25 °C and 37 °C. As in some cases, the number of observations per lineage was not high enough to ensure adequate statistical results, samples from ^a^ were clustered in two groups for this analysis: strong/moderate and weak/non-biofilm producers. /: Χ^2^ test not possible due to lack of observations in a variable. **^c^** Observed cases regarding lineage and distribution of cases concerning the changes in biofilm production capacity depending on the temperature and ^d^ *p*-values from Χ^2^ test, correlating significant differences between lineages.

Biofilm-Forming Capacity ^a^		Lineage I	Lineage II	Lineage III
Strong	25 °C	15	17	1
	37 °C	7	7	1
Moderate	25 °C	0/8	1/10	1/1
	37 °C	8	10	1
Weak	25 °C	0	0	0
	37 °C	1	1	0
Non-biofilm	25 °C	2	0	0
	37 °C	1	0	0
**25 °C**		**Lineage I**	**Lineage II**	
Lineage I				
Lineage II		0.4918 ^b^		
Lineage III		0.6295 ^b^	/	
**37 °C**		**Lineage I**	**Lineage II**	
Lineage I				
Lineage II		0.5119 ^b^		
Lineage III		0.8767 ^b^	/	
**Changes in biofilm-forming capacity ^c^**		**Lineage I**	**Lineage II**	**Lineage III**
Remained strong		7	7	1
Strong to moderate		8	9	0
Strong to weak		0	1	1
Remained moderate		0	1	0
Non-biofilm to weak		1	0	0
remained non-biofilm		1	0	0
		**Lineage I**	**Lineage II**	
**Lineage I**				
**Lineage II**		0.5409 ^d^		
**Lineage III**		0.3403 ^d^	0.1107 ^d^	

**Table 9:** Profiles for antibiotic resistance. Bacterial strains were classified as resistant (R), intermediate resistant (I), or susceptible (S) to the tested antibiotics. The antibiotic analyzed were: amoxicillin + clavulanic (AMC), ampicillin (AMP), amoxicillin (AMX), chloramphenicol (CHL), ciprofloxacin (CIP), clindamycin (CLI), cefotaxime (CTX), erythromycin (ERY), fosfomycin (FOS), fusidic acid (FUS), gentamicin (GEN), imipenem (IPM), kanamycin (KAN), nalidixic acid (NAL), oxacylin (OXA), Bencylpenicillin (PCG), penicillin (PEN), rifampicin (RIF), sulfonamide (SUL), trimethoprim-sulfamethoxazole (SXT), tetracycline (TET), Trimetoprim (TMP) and vancomycin (VAN). *L. m*: *Listeria monocytogenes*; *S. a*: *Staphylococcus aureus*.

Strain	Species	AMC	AMP	AMX	CHL	CIP	CLI	CTX	ERY	FOS	FUS	GEN	IPM	KAN	NAL	OXA	PCG	PEN	RIF	SUL	SXT	TET	TMP	VAN
LMO391	*L. m.*	I	S	S	S	I	R	R	S	R	R	I	S	S	R	R	R	S	I	S	S	S	S	S
LMO392	*L. m*	I	S	S	S	I	R	R	S	R	R	I	S	S	R	R	R	S	S	S	S	S	S	S
LMO393	*L. m*	I	S	S	S	I	R	R	S	R	R	S	S	S	R	R	S	S	S	S	S	S	S	S
LMO394	*L. m*	I	S	S	S	I	R	R	S	R	R	I	S	S	R	R	R	S	I	S	S	S	S	S
LMO395	*L. m*	I	S	S	S	I	R	R	S	R	R	S	S	S	R	R	R	S	I	S	S	S	S	S
LMO396	*L. m*	I	S	S	S	I	R	R	S	R	R	I	S	S	R	R	R	S	I	S	S	S	S	S
LMO397	*L. m*	I	S	S	S	I	S	R	S	R	R	S	S	S	R	R	R	S	S	S	S	S	S	S
LMO398	*L. m*	I	S	S	S	I	R	R	S	R	R	S	S	S	R	R	S	S	I	S	S	S	S	S
LMO399	*L. m*	I	S	S	S	I	R	R	S	R	R	S	S	S	R	R	S	S	S	S	S	S	S	S
LMO400	*L. m*	I	S	S	S	I	R	R	S	R	R	S	S	S	R	R	S	S	I	S	S	S	S	S
LMO401	*L. m*	I	S	S	S	I	R	R	S	R	R	S	S	S	R	R	I	S	I	S	S	S	S	S
LMO402	*L. m*	I	S	S	S	I	R	R	S	R	R	S	S	S	R	R	S	S	S	S	S	S	S	S
LMO403	*L. m*	I	S	S	S	I	R	R	S	R	R	S	S	S	R	R	S	S	I	S	S	S	S	S
LMO404	*L. m*	I	S	S	S	I	R	R	S	R	R	S	S	S	R	R	S	S	I	S	S	S	S	S
LMO405	*L. m*	I	S	S	S	I	R	R	S	R	R	S	S	S	R	R	S	S	S	S	S	S	S	S
LMO406	*L. m*	I	S	S	S	I	R	R	S	R	R	S	S	S	R	R	S	S	S	S	S	S	S	S
LMO407	*L. m*	I	S	S	S	I	R	R	S	S	R	S	S	S	R	R	S	S	S	S	S	S	S	S
LMO408	*L. m*	I	S	S	S	I	R	R	S	R	R	S	S	S	R	R	S	S	S	S	S	S	S	S
LMO409	*L. m*	I	S	S	S	I	R	R	S	R	R	S	S	S	R	R	S	S	S	S	S	S	S	S
LMO410	*L. m*	I	S	S	S	I	R	R	S	R	R	S	S	S	R	R	S	S	S	S	S	S	S	S
LMO411	*L. m*	I	S	S	S	I	R	R	S	R	R	S	S	S	R	R	R	S	S	S	S	S	S	S
LMO412	*L. m*	I	S	S	S	I	R	R	S	R	R	S	S	S	R	R	S	S	S	S	S	S	S	S
LMO413	*L. m*	I	S	S	S	I	R	R	S	R	R	S	S	S	R	R	S	S	S	S	S	S	S	S
LMO414	*L. m*	I	S	S	S	I	R	R	S	R	R	S	S	S	R	R	S	S	S	S	S	S	S	S
LMO415	*L. m*	I	S	S	S	I	R	R	S	R	R	S	S	S	R	R	S	S	S	S	S	S	S	S
LMO416	*L. m*	I	S	S	S	I	R	R	S	R	R	S	S	S	R	R	S	S	I	S	S	S	S	S
LMO417	*L. m*	I	S	S	S	I	R	R	S	R	R	S	S	S	R	R	S	S	I	S	S	S	S	S
LMO418	*L. m*	I	S	S	S	I	R	R	S	R	R	S	S	S	R	R	S	S	I	S	S	S	S	S
LMO419	*L. m*	I	S	S	S	I	R	R	S	R	R	S	S	S	R	R	S	S	S	S	S	S	S	S
LMO420	*L. m*	I	S	S	S	I	R	R	S	R	R	I	S	S	R	R	S	S	I	S	S	S	S	S
LMO421	*L. m*	I	S	S	S	I	S	R	S	R	R	S	S	S	R	R	S	S	I	S	S	S	S	R
LMO422	*L. m*	I	S	S	S	I	R	R	S	R	R	I	S	S	R	R	S	S	I	S	S	S	S	S
LMO423	*L. m*	I	S	S	S	I	R	R	S	R	R	S	S	S	R	R	S	S	S	S	S	S	S	S
LMO424	*L. m*	I	S	S	S	I	R	R	S	R	R	S	S	S	R	R	S	S	S	S	S	S	S	S
LMO425	*L. m*	I	S	S	S	I	R	R	S	R	R	S	S	S	R	R	S	S	I	S	S	S	S	S
LMO426	*L. m*	I	S	S	S	I	R	R	S	R	R	S	S	S	R	R	S	S	I	S	S	S	S	S
LMO428	*L. m*	I	S	S	S	I	R	R	S	R	R	S	S	S	R	R	S	S	S	S	S	S	S	S
ATCC 25923	*S. a*	I	S	S	S	I	S	S	S	S	S	S	S	S	R	S	S	S	S	S	R	S	S	S

**Table 10:** Antimicrobial resistance (AMR) profiles observed in this study and obtained from literature. The number of strains used in each study is indicated, as well as the percentage of strains that shared the same AMR profile.

Reference	Multi-Resistance Profile	Strains	Percentage
This study	AMC^I^ CIP^I^ CTX^R^ FUS^R^ NAL^R^ OXA^R^	37	100.00%
	AMC^I^ CIP^I^ CLI^R^ CTX^R^ FUS^R^ NAL^R^ OXA^R^	35	94.59%
	AMC^I^ CIP^I^ CLI^R^ CTX^R^ FOS^R^ FUS^R^ NAL^R^ OXA^R^	34	91.89%
	AMC^I^ CIP^I^ CLI^R^ CTX^R^ FOS^R^ FUS^R^ NAL^R^ OXA^R^ RIF^I^	17	45.95%
	AMC^I^ CIP^I^ CLI^R^ CTX^R^ FOS^R^ FUS^R^ NAL^R^ OXA^R^ PCG^R^	7	18.92%
	AMC^I^ CIP^I^ CLI^R^ CTX^R^ FUS^R^ GEN^I^ NAL^R^ OXA^R^	6	16.22%
[13]	Resistance not observed	80	65.04%
	Resistance to at least one antibiotic	43	34.96%
	TET^R^	22	17.89%
	PEN^R^	21	17.07%
	SXT^R^	15	12.20%
	SXT^R^ TET^R^	8	6.50%
[43]	AMP^R^	50	71.43%
	Resistance not observed	11	15.71%
	AMC^R^ AMP^R^	3	4.29%
[12]	FUS^R^ PCG^R^	16	100.00%
	FOS^R^ FUS^R^ PCG^R^	14	87.50%
	FUS^R^ OXA^R^ PCG^R^	14	87.50%
	FOS^R^ FUS^R^ OXA^R^ PCG^R^	13	81.25%
	CLI^R^ FOS^R^ FUS^R^ OXA^R^ PCG^R^	10	62.50%
	CIP^R^ FOS^R^ FUS^R^ PCG^R^	8	50.00%
	CIP^R^ CLI^R^ FOS^R^ FUS^R^ PCG^R^	6	37.50%
	CIP^R^ CLI^R^ FOS^R^ FUS^R^ OXA^R^ PCG^R^	5	31.25%
	CLI^R^ FOS^R^ FUS^R^ IPM^R^ OXA^R^ PCG^R^	5	31.25%
[36]	Resistance not observed	25	60.98%
	CLI^R^	7	17.07%
	OXA^R^	7	17.07%
	TETR	4	9.76%
	ERY^R^ OXA^R^	2	4.88%
	ERY^R^ TET^R^	2	4.88%
	ERY^R^ OXA^R^ PEN^R^	1	2.44%
	ERY^R^ OXA^R^ TET^R^	1	2.44%
[11]	NAL^R^	89	100.00%
	NAL^R^ SUL^R^	76	85.39%
	CTX^R^ NAL^R^	57	64.04%
	CTX^R^ NAL^R^ SUL^R^	48	53.93%
	FOS^R^ NAL^R^	45	50.56%
	FOS^R^ NAL^R^ SUL^R^	39	43.82%
	CTX^R^ FOS^R^ NAL^R^ SUL^R^	21	23.60%

**Table 11:** Resistance of *L. monocytogenes* to different antibiotic families considering the results from this study and the extant literature. The table shows the number of isolates resistant to each antibiotic family. In addition, the percentage of strains that showed resistance to each antibiotic family is indicated, both in this study and the literature. N.A.: not analyzed.

		Literature					Percentage	
	This Study	[13]	[43]	[12]	[36]	[11]	This Study	Literature
ß-lactams	37	22	59	16	7	0	100.00%	30.59%
Lincosamides	37	8	0	14	7	1	100.00%	8.82%
Cephalosporins	37	N.A.	N.A.	N.A.	N.A.	57	100.00%	64.04%
Steroids	37	N.A.	N.A.	16	N.A.	0	100.00%	15.24%
Quinolones	37	N.A.	N.A.	N.A.	N.A.	89	100.00%	100.00%
Phosphonic acid derivatives	36	N.A.	N.A.	14	N.A.	46	97.30%	51.69%
Ansamycins	17	N.A.	N.A.	6	N.A.	0	45.95%	5.71%
Aminoglycosides	6	N.A.	0	0	N.A.	0	16.22%	0.00%
Tetracyclines	0	22	0	5	4	N.A.	0.00%	12.35%
Sulfonamides	0	14	0	5	N.A.	76	0.00%	31.88%
Macrolides	0	3	0	0	3	1	0.00%	2.06%
**Number of strains**	37	123	70	16	42	89		

## Data Availability

The data presented in this study are available on request from the main author (famarita@azti.es) because of privacy restrictions.

## Supplementary Materials

The following supporting information can be downloaded at: https://www.mdpi.com/article/10.3390/foods14091580/s1, Table S1: Biofilm formation by *Listeria monocytogenes* isolates at 25 °C by crystal violet staining. OD_C_ is the cut-off optical density value. Strains were classified as non-biofilm producers (OD ≤ OD_C_), weak biofilm producers (OD_C_ < OD ≤ 2× OD_C_), moderate biofilm producers (2× OD_C_ < OD ≤ 4× OD_C_), and strong biofilm producers (4× OD_C_ > OD). Table S2: Biofilm formation by *Listeria monocytogenes* isolates at 37 °C by crystal violet staining. OD_C_ is the cut-off optical density value. Strains were classified as non-biofilm producers (OD ≤ OD_C_), weak biofilm producers (OD_C_ < OD ≤ 2× OD_C_), moderate biofilm producers (2× OD_C_ < OD ≤ 4× OD_C_), and strong biofilm producers (4× OD_C_ > OD); Table S3: Biofilm formation by other Listeria spp. isolates at 25 °C by crystal violet staining. ODC is the cut-off optical density value. Strains were classified as non-biofilm producers (OD ≤ ODC), weak biofilm producers (ODC < OD ≤ 2× ODC), moderate biofilm producers (2× ODC < OD ≤ 4× ODC), and strong biofilm producers (4× ODC > OD); Table S4: Biofilm formation by other Listeria spp. isolates at 37 °C by crystal violet staining. ODC is the cut-off optical density value. Strains were classified as non-biofilm producers (OD ≤ ODC), weak biofilm producers (ODC < OD ≤ 2× ODC), moderate biofilm producers (2× ODC < OD ≤ 4× ODC), and strong biofilm producers (4× ODC > OD).
