# Supplementary material for: Genetic Diversity, Biofilm Formation, and Antibiotic Resistance in Listeria monocytogenes Isolated from Meat-Processing Plants
Source: Foods. 2025 Apr 30;14(9):1580. doi: 10.3390/foods14091580 (PMC12071657; doi:10.3390/foods14091580)
Supplement: Supplementary file 1 [file foods-14-01580-s001.zip › foods-3582483-supplementary.pdf]

Table S1. Biofilm formation by *Listeria monocytogenes* isolates at 25 ºC by crystal violet staining. OD_C is the cut-off optical density value. Strains were classified as non-biofilm producers ($OD \leq OD_C$), weak biofilm producers ($OD_C < OD \leq 2 \times OD_C$), moderate biofilm producers ($2 \times OD_C < OD \leq 4 \times OD_C$), and strong biofilm producers ($4 \times OD_C > OD$).

Strain \ OD_{600}	1	2	3	4	5	Mean	SD	Mean-Acetic	Biofilm forming capacity
LMO391	0.336	0.263	0.230	0.239	0.211	0.256	0.049	0.108	Strong
LMO392	0.269	0.244	0.265	0.202	0.226	0.241	0.028	0.094	Strong
LMO393	0.249	0.252	0.242	0.242	0.196	0.236	0.023	0.089	Strong
LMO394	0.490	0.412	0.446	0.460	0.357	0.433	0.051	0.286	Strong
LMO395	0.200	0.226	0.256	0.168	0.167	0.203	0.038	0.056	Strong
LMO396	0.204	0.265	0.252	0.225	0.198	0.229	0.029	0.081	Strong
LMO397	0.355	0.437	0.365	0.370	0.301	0.366	0.048	0.218	Strong
LMO398	0.309	0.317	0.264	0.278	0.246	0.283	0.030	0.135	Strong
LMO399	0.385	0.334	0.329	0.335	0.346	0.346	0.023	0.198	Strong
LMO400	0.107	0.152	0.101	0.101	0.102	0.113	0.022	-0.035	Non-Biofilm
LMO401	0.255	0.284	0.217	0.247	0.250	0.251	0.024	0.103	Strong
LMO402	0.311	0.282	0.238	0.298	0.272	0.280	0.028	0.133	Strong
LMO403	0.237	0.235	0.212	0.207	0.216	0.221	0.014	0.074	Strong
LMO404	0.236	0.228	0.211	0.204	0.207	0.217	0.014	0.070	Strong
LMO405	0.237	0.259	0.206	0.217	0.249	0.234	0.022	0.086	Strong
LMO406	0.433	0.396	0.385	0.350	0.325	0.378	0.042	0.230	Strong
LMO407	0.193	0.205	0.166	0.244	0.235	0.209	0.032	0.061	Strong
LMO408	0.309	0.304	0.247	0.260	0.291	0.282	0.027	0.135	Strong
LMO409	0.234	0.240	0.184	0.351	0.282	0.258	0.062	0.111	Strong
LMO410	0.191	0.181	0.173	0.199	0.166	0.182	0.013	0.035	Moderate
LMO411	0.231	0.307	0.289	0.188	0.288	0.261	0.050	0.113	Strong
LMO412	0.226	0.294	0.300	0.273	0.242	0.267	0.032	0.120	Strong
LMO413	0.253	0.272	0.246	0.251	0.289	0.262	0.018	0.115	Strong
LMO414	0.249	0.285	0.304	0.238	0.289	0.273	0.028	0.126	Strong
LMO415	0.203	0.274	0.233	0.228	0.272	0.242	0.031	0.095	Strong
LMO416	0.246	0.279	0.283	0.226	0.270	0.261	0.024	0.113	Strong
LMO417	0.216	0.217	0.201	0.222	0.255	0.222	0.020	0.075	Strong
LMO418	0.361	0.319	0.270	0.300	0.312	0.312	0.033	0.165	Strong
LMO419	0.280	0.252	0.252	0.257	0.291	0.266	0.018	0.119	Strong
LMO420	0.227	0.260	0.235	0.251	0.254	0.245	0.014	0.098	Strong
LMO421	0.375	0.323	0.319	0.255	0.333	0.321	0.043	0.174	Strong
LMO422	0.213	0.238	0.225	0.278	0.274	0.246	0.029	0.098	Strong
LMO423	0.257	0.169	0.181	0.244	0.265	0.223	0.045	0.076	Strong
LMO424	0.209	0.205	0.233	0.330	0.331	0.262	0.064	0.114	Strong
LMO425	0.167	0.162	0.146	0.162	0.150	0.157	0.009	0.010	Non-Biofilm
LMO426	0.192	0.193	0.184	0.151	0.185	0.181	0.017	0.034	Moderate
LMO427	0.184	0.205	0.192	0.168	0.186	0.187	0.013	0.040	Moderate
LMO428	0.357	0.330	0.283	0.230	0.253	0.291	0.053	0.143	Strong
Neg Control	0.150	0.152	0.144	0.151	0.147	0.149	0.003	0.004	
Acetic acid	0.168	0.150	0.147	0.168	0.104	0.147	0.026	0.011	ODc

Table S2. Biofilm formation by *Listeria monocytogenes* isolates at 37 ºC by crystal violet staining. OD_C is the cut-off optical density value. Strains were classified as non-biofilm producers ($OD \leq OD_C$), weak biofilm producers ($OD_C < OD \leq 2 \times OD_C$), moderate biofilm producers ($2 \times OD_C < OD \leq 4 \times OD_C$), and strong biofilm producers ($4 \times OD_C > OD$).

Strain \ OD_{600}	1	2	3	4	5	Mean	SD	Mean-Acetic	Biofilm forming capacity
LMO391	0.367	0.311	0.327	0.499	0.368	0.374	0.074	0.215	Moderate
LMO392	0.301	0.370	0.319	0.342	0.363	0.339	0.029	0.179	Moderate
LMO393	0.383	0.405	0.378	0.302	0.385	0.371	0.040	0.211	Moderate
LMO394	0.353	0.507	0.416	0.341	0.409	0.405	0.066	0.245	Moderate
LMO395	0.261	0.333	0.364	0.321	0.365	0.329	0.042	0.169	Moderate
LMO396	0.313	0.424	0.281	0.389	0.335	0.348	0.058	0.189	Moderate
LMO397	0.545	0.438	0.412	0.369	0.598	0.472	0.096	0.313	Strong
LMO398	0.577	0.624	0.477	0.356	0.558	0.518	0.105	0.359	Strong
LMO399	0.430	0.369	0.476	0.373	0.392	0.408	0.045	0.248	Moderate
LMO400	0.103	0.102	0.102	0.139	0.110	0.111	0.016	-0.049	Non-Biofilm
LMO401	0.613	0.647	0.451	0.501	0.364	0.515	0.116	0.355	Strong
LMO402	0.434	0.414	0.365	0.446	0.482	0.428	0.043	0.268	Moderate
LMO403	0.405	0.428	0.502	0.510	0.479	0.465	0.046	0.305	Strong
LMO404	0.440	0.525	0.484	0.416	0.445	0.462	0.043	0.302	Strong
LMO405	0.536	0.658	0.507	0.548	0.599	0.570	0.060	0.410	Strong
LMO406	0.525	0.599	0.589	0.444	0.412	0.514	0.084	0.354	Strong
LMO407	0.257	0.305	0.305	0.264	0.253	0.277	0.026	0.117	Weak
LMO408	0.574	0.694	0.649	0.608	0.632	0.631	0.045	0.472	Strong
LMO409	0.704	0.634	0.584	0.568	0.622	0.622	0.053	0.463	Strong
LMO410	0.393	0.364	0.298	0.304	0.325	0.337	0.041	0.177	Moderate
LMO411	0.521	0.398	0.383	0.359	0.456	0.423	0.065	0.264	Moderate
LMO412	0.472	0.403	0.568	0.359	0.461	0.453	0.079	0.293	Strong
LMO413	0.488	0.393	0.617	0.522	0.516	0.507	0.080	0.347	Strong
LMO414	0.408	0.409	0.417	0.324	0.339	0.379	0.044	0.220	Moderate
LMO415	0.473	0.543	0.501	0.471	0.388	0.475	0.057	0.315	Strong
LMO416	0.503	0.352	0.344	0.342	0.428	0.394	0.071	0.234	Moderate
LMO417	0.597	0.337	0.384	0.378	0.327	0.405	0.110	0.245	Moderate
LMO418	0.684	0.541	0.550	0.549	0.632	0.591	0.064	0.431	Strong
LMO419	0.667	0.406	0.481	0.346	0.465	0.473	0.121	0.313	Strong
LMO420	0.458	0.399	0.340	0.422	0.366	0.397	0.046	0.237	Moderate
LMO421	0.498	0.364	0.386	0.362	0.336	0.389	0.063	0.229	Moderate
LMO422	0.411	0.530	0.405	0.443	0.385	0.435	0.057	0.275	Strong
LMO423	0.373	0.358	0.350	0.343	0.411	0.367	0.027	0.207	Moderate
LMO424	0.482	0.377	0.383	0.397	0.484	0.425	0.054	0.265	Moderate
LMO425	0.265	0.280	0.243	0.279	0.369	0.287	0.048	0.127	Weak
LMO426	0.392	0.384	0.325	0.386	0.378	0.373	0.027	0.213	Moderate
LMO427	0.290	0.392	0.321	0.366	0.391	0.352	0.045	0.192	Moderate
LMO428	0.416	0.331	0.308	0.432	0.359	0.369	0.053	0.209	Moderate
Neg Control	0.161	0.179	0.174	0.205	0.167	0.177	0.017	0.017	
Acetic acid	0.187	0.181	0.167	0.164	0.100	0.160	0.035	0.068	ODc

Table S3. Biofilm formation by other *Listeria* spp. isolates at 25 ºC by crystal violet staining. OD_C is the cut-off optical density value. Strains were classified as non-biofilm producers ($OD \leq OD_C$), weak biofilm producers ($OD_C < OD \leq 2 \times OD_C$), moderate biofilm producers ($2 \times OD_C < OD \leq 4 \times OD_C$), and strong biofilm producers ($4 \times OD_C > OD$).

Strain \ OD_{600}	1	2	3	4	5	Mean	SD	Mean-Acetic	Biofilm forming capacity
LIN014	0.201	0.184	0.200	0.197	0.173	0.191	0.012	0.037	Non-Biofilm
LIN015	0.191	0.186	0.193	0.198	0.174	0.188	0.009	0.034	Non-Biofilm
LIN016	0.196	0.193	0.177	0.184	0.194	0.189	0.008	0.035	Non-Biofilm
LIN017	0.179	0.182	0.184	0.182	0.180	0.181	0.002	0.027	Non-Biofilm
LIN018	0.178	0.175	0.180	0.189	0.175	0.179	0.006	0.025	Non-Biofilm
LIN019	0.190	0.172	0.164	0.180	0.166	0.174	0.011	0.020	Non-Biofilm
LIN020	0.231	0.225	0.205	0.215	0.221	0.219	0.010	0.065	Non-Biofilm
LIN021	0.211	0.204	0.170	0.202	0.181	0.194	0.017	0.040	Non-Biofilm
LIN022	0.229	0.221	0.209	0.207	0.213	0.216	0.009	0.062	Non-Biofilm
LIN023	0.181	0.184	0.155	0.178	0.176	0.175	0.011	0.021	Non-Biofilm
LIN024	0.204	0.187	0.201	0.205	0.199	0.199	0.007	0.045	Non-Biofilm
LIN025	0.210	0.200	0.198	0.188	0.177	0.195	0.013	0.041	Non-Biofilm
LIN026	0.155	0.147	0.144	0.145	0.147	0.148	0.004	-0.006	Non-Biofilm
LIN027	0.187	0.173	0.141	0.166	0.175	0.168	0.017	0.014	Non-Biofilm
LIN028	0.160	0.146	0.155	0.162	0.163	0.157	0.007	0.003	Non-Biofilm
LIN029	0.152	0.159	0.152	0.153	0.153	0.154	0.003	0.000	Non-Biofilm
LIN030	0.170	0.183	0.204	0.220	0.220	0.199	0.022	0.045	Non-Biofilm
LIN031	0.172	0.202	0.207	0.244	0.233	0.212	0.028	0.058	Non-Biofilm
LIN032	0.178	0.167	0.168	0.196	0.169	0.176	0.012	0.022	Non-Biofilm
LWE002	0.155	0.158	0.150	0.158	0.151	0.154	0.004	0.000	Non-Biofilm
LWE003	0.163	0.145	0.152	0.174	0.169	0.161	0.012	0.007	Non-Biofilm
LWE005	0.148	0.132	0.136	0.154	0.131	0.140	0.010	-0.014	Non-Biofilm
LWE006	0.168	0.148	0.144	0.153	0.137	0.150	0.012	-0.004	Non-Biofilm
LWE007	0.169	0.142	0.151	0.171	0.144	0.155	0.014	0.001	Non-Biofilm
LWE008	0.180	0.160	0.164	0.177	0.148	0.166	0.013	0.012	Non-Biofilm
LWE009	0.170	0.149	0.150	0.159	0.158	0.157	0.008	0.003	Non-Biofilm
LWE010	0.156	0.142	0.134	0.141	0.138	0.142	0.008	-0.012	Non-Biofilm
LWE011	0.154	0.135	0.142	0.149	0.149	0.146	0.007	-0.008	Non-Biofilm
LWE012	0.155	0.162	0.149	0.155	0.140	0.152	0.008	-0.002	Non-Biofilm
LWE013	0.161	0.163	0.165	0.193	0.189	0.174	0.015	0.020	Non-Biofilm
LWE014	0.304	0.270	0.289	0.294	0.272	0.286	0.015	0.132	Weak
LWE015	0.188	0.168	0.160	0.178	0.155	0.170	0.013	0.016	Non-Biofilm
LWE016	0.216	0.198	0.196	0.221	0.206	0.207	0.011	0.053	Non-Biofilm
LWE017	0.175	0.167	0.152	0.158	0.167	0.164	0.009	0.010	Non-Biofilm
LWE018	0.233	0.177	0.166	0.182	0.182	0.188	0.026	0.034	Non-Biofilm
LWE019	0.246	0.181	0.195	0.171	0.171	0.193	0.031	0.039	Non-Biofilm
LWE020	0.236	0.194	0.178	0.174	0.182	0.193	0.025	0.039	Non-Biofilm
LWE021	0.266	0.215	0.176	0.249	0.198	0.221	0.037	0.067	Weak
Neg Control	0.135	0.151	0.143	0.188	0.167	0.157	0.021	0.003	
Acetic acid	0.134	0.160	0.151	0.149	0.176	0.154	0.015	0.066	ODc

Table S4. Biofilm formation by other *Listeria* spp. isolates at 37 ºC by crystal violet staining. OD_C is the cut-off optical density value. Strains were classified as non-biofilm producers ($OD \leq OD_C$), weak biofilm producers ($OD_C < OD \leq 2 \times OD_C$), moderate biofilm producers ($2 \times OD_C < OD \leq 4 \times OD_C$), and strong biofilm producers ($4 \times OD_C > OD$).

Strain \ OD_{600}	1	2	3	4	5	Mean	SD	Mean-Acetic	Biofilm forming capacity
LIN014	0.362	0.344	0.321	0.432	0.392	0.370	0.043	0.206	Strong
LIN015	0.436	0.402	0.410	0.410	0.419	0.415	0.013	0.251	Strong
LIN016	0.351	0.348	0.346	0.345	0.346	0.347	0.002	0.183	Moderate
LIN017	0.358	0.346	0.329	0.319	0.312	0.333	0.019	0.169	Moderate
LIN018	0.341	0.324	0.369	0.323	0.337	0.339	0.019	0.175	Moderate
LIN019	0.410	0.369	0.376	0.358	0.326	0.368	0.030	0.204	Strong
LIN020	0.306	0.329	0.296	0.267	0.261	0.292	0.028	0.128	Moderate
LIN021	0.375	0.372	0.267	0.263	0.238	0.303	0.065	0.139	Moderate
LIN022	0.346	0.346	0.337	0.319	0.304	0.330	0.018	0.166	Moderate
LIN023	0.324	0.276	0.274	0.263	0.242	0.276	0.030	0.112	Moderate
LIN024	0.396	0.494	0.425	0.431	0.402	0.430	0.039	0.266	Strong
LIN025	0.389	0.318	0.376	0.343	0.358	0.357	0.028	0.193	Strong
LIN026	0.218	0.215	0.216	0.258	0.255	0.232	0.022	0.068	Weak
LIN027	0.202	0.196	0.225	0.223	0.213	0.212	0.013	0.048	Weak
LIN028	0.459	0.399	0.382	0.360	0.350	0.390	0.043	0.226	Strong
LIN029	0.490	0.507	0.461	0.395	0.348	0.440	0.067	0.276	Strong
LIN030	0.213	0.230	0.212	0.262	0.215	0.226	0.021	0.062	Weak
LIN031	0.397	0.332	0.316	0.331	0.325	0.340	0.032	0.176	Moderate
LIN032	0.238	0.189	0.206	0.234	0.218	0.217	0.020	0.053	Weak
LWE002	0.239	0.241	0.219	0.228	0.224	0.230	0.010	0.066	Weak
LWE003	0.267	0.267	0.201	0.196	0.204	0.227	0.037	0.063	Weak
LWE005	0.287	0.245	0.237	0.244	0.237	0.250	0.021	0.086	Weak
LWE006	0.252	0.239	0.207	0.230	0.192	0.224	0.024	0.060	Weak
LWE007	0.195	0.167	0.164	0.173	0.161	0.172	0.014	0.008	Non-Biofilm
LWE008	0.276	0.251	0.226	0.214	0.197	0.233	0.031	0.069	Weak
LWE009	0.206	0.176	0.174	0.177	0.159	0.178	0.017	0.014	Non-Biofilm
LWE010	0.220	0.186	0.173	0.199	0.151	0.186	0.026	0.022	Non-Biofilm
LWE011	0.190	0.166	0.159	0.199	0.174	0.178	0.017	0.014	Non-Biofilm
LWE012	0.254	0.255	0.236	0.266	0.227	0.248	0.016	0.084	Weak
LWE013	0.312	0.260	0.259	0.250	0.258	0.268	0.025	0.104	Moderate
LWE014	0.323	0.266	0.245	0.231	0.229	0.259	0.039	0.095	Moderate
LWE015	0.386	0.329	0.244	0.258	0.205	0.284	0.072	0.120	Moderate
LWE016	0.303	0.309	0.245	0.315	0.236	0.282	0.038	0.118	Moderate
LWE017	0.360	0.385	0.243	0.237	0.253	0.296	0.071	0.132	Moderate
LWE018	0.320	0.234	0.268	0.245	0.266	0.267	0.033	0.103	Moderate
LWE019	0.400	0.417	0.285	0.242	0.233	0.315	0.087	0.151	Moderate
LWE020	0.365	0.480	0.299	0.283	0.279	0.341	0.085	0.177	Moderate
LWE021	0.262	0.315	0.332	0.227	0.201	0.267	0.056	0.103	Moderate
Neg Control	0.184	0.146	0.148	0.138	0.131	0.149	0.020	-0.015	
Acetic acid	0.180	0.164	0.142	0.164	0.170	0.164	0.014	0.047	ODc
